# Resilience and prenatal mental health in Pakistan: a qualitative inquiry

**DOI:** 10.1186/s12884-022-05176-y

**Published:** 2022-11-14

**Authors:** Shireen Shehzad Bhamani, David Arthur, An-Sofie Van Parys, Nicole Letourneau, Gail Wagnild, Shahirose Sadrudin Premji, Nargis Asad, Olivier Degomme

**Affiliations:** 1 School of Nursing and Midwifery, Aga Khan University, Karachi, Pakistan; 2 Ghent University, Ghent, Belgium; 3 Bermi Acupuncture & Chinese Medicine Clinic, Bermagui, NSW Australia; 4 Peking Union Medical College, Beijing, China; 5 University of Calgary, Calgary, Canada; 6 Resilience Center, Montana, USA; 7 School of Nursing, Faculty of Health, York University, Ontario, Canada; 8 Department of Psychiatry, Aga Khan University, Karachi, Pakistan

**Keywords:** Resilience, Pregnant women, Perinatal mental illness, Depression

## Abstract

**Background:**

Women in Pakistan suffer from a high rate of depression. The stress of low-income, illiteracy, exposure to violence and living in a patriarchal society are predisposing vulnerabilities for depression, particularly during and following pregnancy. The resilience of an individual plays a significant role in promoting prenatal mental health, but this has yet to be thoroughly researched. In this article, our objective is to identify the core characteristics of resilience among pregnant women, which will then help us in developing an intervention.

**Methods:**

The exploratory-descriptive study was conducted over 6 months in five different antenatal hospitals in Sindh, Pakistan. A total of 17 semi-structured interviews were conducted with pregnant women, purposefully selected with heterogeneous characteristics to explore diverse perspectives, while symptoms of depression were quantified by the Edinburgh Postnatal Depression Scale before the interview. Verbatim transcriptions were coded openly and merged into categories and themes.

**Result:**

A total of six themes emerged from in-depth thematic analysis: 1) purpose of life, 2) dealing with emotions, 3) believing in yourself, 4) optimistic approach, 5) strengthening support and relationship and 6) spirituality and humanity. Women agreed that these characteristics could help them improve their mental health.

**Conclusion:**

In conclusion, these themes were the core components of pregnant women’s resilience which ultimately could help to promote prenatal mental health. These pave a pathway towards developing culturally and contextually resilience interventions aimed at enhancing mental health of pregnant women which then may improve neonatal and family mental wellbeing.

## Background

Depression during pregnancy is a global concern [1, 2] complicated by associated socio-demographic factors such as income, education, age, personal history and pregnancy complications [3]. Ongoing negative outcomes include maternal mortality and compromised child health [4]. Studies conducted in low- and middle-income countries (LMICs) reveal symptoms of depression ranging from 12.7 to 37% at some point during pregnancy [2, 5–8]. In a rural sub district of Pakistan where 1154 pregnant women were sampled, the prevalence of depression was over 26%, [9].

In LMICs, predictors of prenatal mental illness including depression consist of financial constraints, stress regarding the sex of the baby, conflicts with mother-in-law or husband, and poor family support [10]. Other studies point to the stress of low-income, illiteracy, exposure to violence and living in a patriarchal society as key factors increasing vulnerability to depression [11–16]. In Pakistan, where depression is highly prevalent, various pervasive risk factors increase the likelihood of mental illness, such as lack of economic and social support, being an unemployed woman, lack of education and domestic abuse [17, 18]. In a broader context, societal norms, socioeconomic environment, and cultural taboos all have a substantial impact on the mental health of pregnant women [19]. Hence, it is evident that the predictors vary from an individual to a societal level, which increase vulnerability to depression [20].

Depression during pregnancy can have repercussions in later life for the mother and present as eating disorders, post-traumatic stress disorder, and personality disorders [21]. Furthermore, despite the high prevalence of depression in low- and middle-income countries, mental health management is stigmatized [22–24]. Such stigma may compound lower self-esteem issues in these women, and decrease their help seeking behaviour [25]. Depression during pregnancy can also negatively impact child health outcomes. In Pakistan, low birth weight, growth retardation, and delayed cognitive and motor development are some of the outcomes associated with children of depressed mothers [26, 27].

Perinatal care to ensure safe pregnancy and childbirth planning has a positive impact on health-seeking behavior and it reduces the risk of illness during pregnancy [28]. Prenatal mental health care is an important component of this yet limited mental health resources or lack of availability of health care facilities make it difficult for women to seek appropriate mental health care. Exploring resilience during pregnancy may help to better understand how to promote Pakistani women’s mental health throughout pregnancy. Individual’s resilience, which is defined as one’s ability to cope with a stressful situation, can function to reduce the risk of mental illness [29] including depression in pregnant women. Interestingly, pregnancy is a period during which resilience extends from the individual level and becomes of particular importance when considering the impact and the woman’s role in family and society [30]. The context surrounding pregnancy can either act as a protective shield or contribute towards stress because social and familial support can help with coping, the social and family environment during pregnancy can operate as a protective factor. In a similar way, the pregnant woman may be more vulnerable if there is no supportive environment [30, 31]. According to the resilience governance framework, resilience is the capacity for adaptation, absorption, and transformation related to a stressful event [32]. In other words, resilient people are able to manage stress and reduce symptoms of depression [33], enhancing positivity which enables an individual to think freely, creatively, and solve problems; hence manage the stressful situation and pregnancy process effectively and in a healthy way [34, 35].

A study conducted in Europe with 151 pregnant women identified that those who were resilient scored higher on self-acceptance, had greater psychological wellbeing, and rated lower levels of pregnancy related stress and postpartum depression, as compared to those who scored low in resilience [36]. Similarly, other studies done in LMIC have also concluded that resilience factors act as a buffer and coping mechanism against stress and depression [3, 37]. Moreover, women may possess a significant capacity to care for their family and the home. The role of a woman is a collection of responsibilities that she may perform to provide resources for her husband, children and others [38]. A meta-synthesis of 15 countries found that women living in LMIC are expected to perform major household tasks without any compensation and are constrained from making health decisions in the family [39]. This role has a special significance when a woman is expecting, thus assisting a particular woman in learning good, constructive skills may have an impact on her personal health, family, and community.

Given that resilience has a very broad definition, examining it without a frame of reference is challenging, so the current study will rely on the ecological framework proposed by Bronfenbrenner. Which was strategically organized into four tiers of external impacts (microsystem, mesosystem, exosystem, and macrosystems) [40, 41], Each of these systems can influences behavior and health of an individual [42]. Microsystem refers to individual factors and their interactions; mesosystem refers to the relationship with various settings in which the individual is involved; exosystem refers to forces within the larger social system in which the individual is embedded eg unemployment while macrosystem refers to cultural beliefs and values that can influence other system. Pregnant women will undoubtedly confront societal, cultural, and familial pressures during this time, which fits within the definition of the ecological model and encourage exploration of these systems as component of resilience and enabling the development of a culturally and contextually acceptable health promotion intervention [30, 43].

In vulnerable groups of pregnant woman, with appropriate screening, the opportunity exists to better understand what enhances resilience with the possibility to improve potential, strength, courage and meaning to life, as these individuals develop new abilities to respond to stress [44, 45]. The most appropriate and practical approach for this study is to explore the attributes of resilience to develop an intervention which can help women in creating their own set of skills and techniques that are immediately and directly applicable to their daily lives throughout their pregnancy to promote mental wellbeing. As there is limited literature reporting on resilience during pregnancy, in either high-income countries or LMICs, the objective of current paper is to identify the core characteristics of the resilience that pregnant women develop which ultimately could help to promote mental health.

## Methods

This is part of a larger project, which will be conducted in a two-phased approach. This paper focuses on phase one and describes the qualitative inquiry to explore the resilience core attributes. These findings will be used to guide the development of resilience building intervention, which will be carried out during the second phase of the larger project using a randomized-controlled trial to evaluate the developed intervention among pregnant women.

### Research design

This qualitative inquiry was conducted using an exploratory- descriptive study design which highlighted the experiences and resilience attributes in a sample of pregnant women in Sindh, Pakistan. Over a period of 6 months from May to October 2019, face-to-face interviews were conducted in the local language Urdu, in a safe private room. The interviews lasted 45–60 minutes and were tape recorded after receiving written informed consent. Observational notes were also made during the interview.

### Study setting

Participants were recruited from the antenatal clinics of the four secondary hospitals of the Aga Khan University Hospital (AKUH) in Karachi, Pakistan. AKUH is a private not for profit institution, serving patients with mixed ethnic and socio-economic backgrounds, from different regions across the country. We also included one other hospital, Koohi Goht (KG) Hospital of Karachi, which provides a midwifery-led service to the most vulnerable women in the community from low socio-economic strata. The rationale for selecting these five settings was purposeful, to ensure a rich database from different socio-economic and ethnic groups, reflecting the cultural diversity that exists across Karachi, Pakistan.

### Sampling strategy and recruitment

Permission was obtained from the heads/representatives of all five sites, staff were informed about the research and written consent was obtained from participants. The purposive sampling technique was used to intentionally select participants for interviews to attain the perspectives of a diverse group of participants from wide-ranging of settings and experiences. Purposive sampling based on criteria allows us to accomplish the study’s objectives while lowering selection bias [46].

Seventeen participants were recruited from the five sites, using the information provided by their head or representatives based on our eligibility criteria. Our eligibility criteria enabled us to obtain a sample that accurately represented the target population. The 13 participants from the four AKU secondary hospitals were initially contacted by telephone for consent. Six of the 17 individuals were also involved in an ongoing study [47]. The remaining four participants from KG were recruited from the waiting area of the hospital. All participants were briefed about the research and similar procedures were followed. After 17 interviews, data collection was stopped due to the repetition of information and confirmation of previously gathered data, which indicated data saturation.

### Eligibility criteria

Participants who were 18 years of age and above, married, at a gestation of 12 weeks and above, and able to speak and understand Urdu were recruited.

### Description of materials

Participants were asked to: 1) complete a demographic questionnaire; 2) complete the Edinburgh Postnatal Depression Scale (EPDS); and 3) engage in a face-to-face semi structured in-depth interview. Depression symptoms were measured by the EPDS Scale, and resilience attributes were explored through interviews. The EPDS has 10 items (each scoring 0–3) with total scores ranging from 0 to 30 and is widely used and shown to be reliable and valid for use in pregnancy [48], and in Urdu [49]. Higher scores indicate greater symptomatology with scores of 10 or more indicating “at risk” for depression, while scores of 13 or more identify depression consistent with a physician diagnosis of major depressive disorder [50–52]. To identify participants with depressive symptoms from mild to serious, 10 was adopted as the cut-off score. The demographic questionnaire included items assessing age, education, language, family (nuclear/extended) type, marriage details and reproductive history.

#### Interview guide

The guide was developed by the research team based on a comprehensive review of the literature, and an existing conceptual framework of resilience underpinning Wagnild and Young’s Resilience Scale [44, 45]. This resilience scale is frequently used in studies about the resilience conducted in Pakistan, and the scale has been validated for use among Pakistani married women [53–58]. The principal investigator (PI) of the current study performed this validation while carefully taken into consideration the content and cultural biases [44] which measures the following five attributes that strengthen an individual’s capacity for resilience: 1): an understanding of the meaning of life; 2) perseverance/determination; 3) existential aloneness/friendship with self; 4) equanimity and 5) self-reliance. The interview guide was then verified by the authors (SSB, DA, AS, NL, SSP, GW and OD) who have vast experience in the field of mental health, perinatal health, and qualitative research. (Refer Table 1).

**Table 1:** Interview Guide

**MEANING OF LIFE**	
• How do you describe your life? Tell us something about your life including the pregnancy phase? • In your opinion, which phase of your life satisfies you the most? • In your opinion what is the purpose of one’s life? • In your opinion what is the purpose of your life? • What do you think are the ways to achieve/fulfill the purpose of your life? • If you get a chance, what would you wish to change in your life? Also give reasons why do you wish to change? • What do you think are the changes you see in your life after marriage and during pregnancy? (Personality differences, life changes, strengths, weaknesses etc)	
**PERSEVERANCE / DETERMINATION**	
• As life is full of ups and downs, In your opinion what is the most difficult or challenging phase of your life? • With reference to what you just told us, have you ever come across any such situation that you successfully overcame? What were the strategies that you used to keep you strong? • With reference to what you just told us, have you come across any such situation where you decided to give up? What were the reasons that made you give up? • According to you, what are the factors that keeps an individual strong?	
**EQUANIMITY**	
• How much do you agree with this statement, “Whatever happens, happens for a reason” • How much do you agree with this statement that “I can always be happy in all situations”	
**EXISTENTIAL ALONENESS/ FRIENDSHIP WITH SELF**	
• What do you like most about yourself? And tell us why? • In your opinion how happy are you with yourself? • In your opinion, how proud you are of yourself? • In your opinion how self-sufficient/independent are you? And if so, why and how? • In your opinion, how do people feel in your presence? OR how do you think you inspire people around you? • In your opinion, how do you feel around people? OR how do people around you inspire you?	
**SELF-RELAINCE**	
• In your opinion what are you good at? (It could be anything) • At what level do you participate in household decision making matters? • Do you think everyone has their own strengths and weaknesses and that if we work hard, we will succeed? (Explain) • How independent you are in making your own decisions?	

### Data analysis

Using thematic analysis, data were manually analyzed from multiple sources including listening to recorded audios, reviewing transcriptions, field notes, and reflections of the researcher to increase validity of data and decrease researcher bias. The research team members that were closely engaged in data analysis and management were SSB, DA and AV.

The following steps were adopted:

1. Use of the bracketing technique in which researchers consciously put aside their biases related to the study before commencing interviews so that the data are collected without judgment [59].
2. Reflective notes were written after each interview and shared and discussed with the research team. This helped in identifying recurrent themes during analysis [59].
3. The interviews were conducted in Urdu and audio recorded, transcribed in Urdu and then translated into English. Back translation into Urdu followed and comparisons were made with the original Urdu transcription to detect any discrepancies in translation. This added validity to the audio recording and transcription process.
4. Open coding was done manually from transcriptions by the research team members. From the participants’ quotes/verbatim, codes were allocated, then related codes were merged or grouped into separate categories, and then associated categories were finally aggregated into one theme.

Hence, one researcher (SSB) initially coded the transcripts. However, throughout data collection and analysis, codes, categories, and themes were created and agreed upon in discussions with the qualitative researchers (AV and DA). They independently coded the transcripts of random interviews and then carefully and constructively examined the initial coding to compare and analyze the results. Interviews proceeded until there were no more new themes emerging from the data or when data saturation was reached. The entire research team shared and reviewed all findings before deciding on the final coding and thematic framework.

## Results

The mean age of participants was 27.9 years (standard deviation (SD) 6.912, range 16 to 39 years). Mean gestational age was 30.8 weeks (SD6.84), rangefrom 16 to 40 weeks). All participants were married (duration ranged from less than 1 to 23 years), and only two were working (one doing office work and the other working from home doing stitching). Despite the diversity of their first languages, which reflected their ethnicity and region of residence, all participants were proficient in Urdu. (Table 2). Notably, nine participants (53%) were rated as having symptoms of depression on the EPDS.

**Table 2:** Socio-Demographic Data

Characteristics	Participants (***N*** = 17)	
	N	%
**Formal Education**		
Yes	15	88
No	2	12
**Education Status**		
No Education	2	12
Primary	1	6
Secondary	3	18
Matriculate	4	23
Intermediate	2	12
Graduation	5	29
**Working Status**		
Yes	2	12
No	15	88
**Type of Marriage**		
Arranged	10	59
Self-Choice	7	41
**Type of Family**		
Nuclear	7	41
Extended	10	59
**Mother tongue**		
Urdu	5	29
Sindhi	4	24
Punjabi	2	12
Gilgiti	1	6
Gujrati	1	6
Pushto	1	6
Saraiki	3	18
**Married within Family**		
Yes	8	47
No	9	53
**Previous Miscarriage**		
Yes	6	35
No	11	65
**Previous Still Birth**		
Yes	1	6
No	16	94
**Alive Child/Children**		
Yes	14	82
No	3	18
**Problem in Current Pregnancy**		
Yes	6	35
No	11	65
**Depressive Symptoms**		
Yes	9	53
No	8	47

**Fig. 1:**
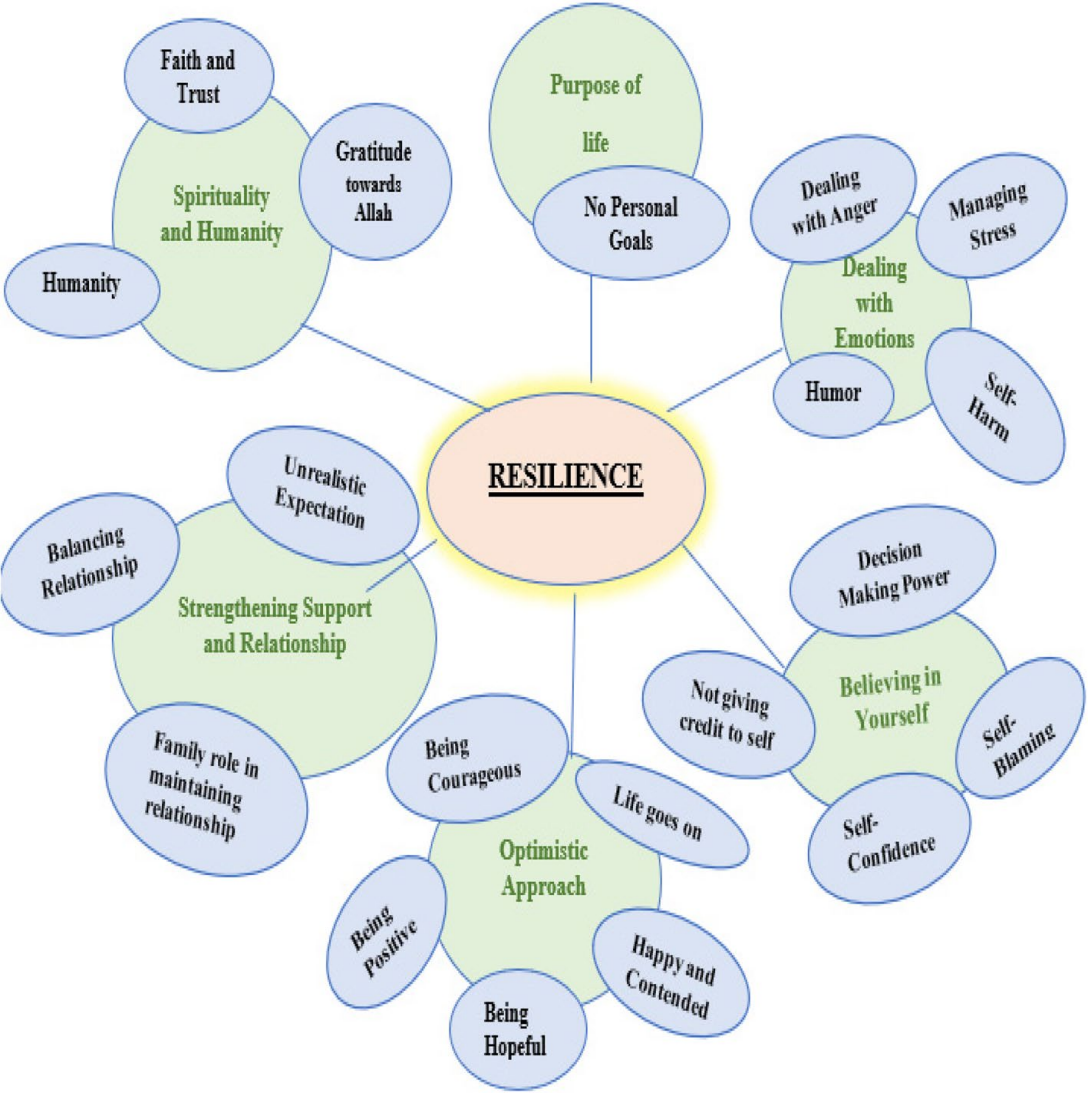
Themes and Categories

Our main qualitative findings identified six themes that emerged from the data which exemplify resilience attributes and included: 1) purpose of life; 2) dealing with emotions; 3) believing in self; 4) optimistic approach; 5) strengthening support and relationship; and 6) spirituality and humanity (Fig. 1).

The following section reports the essence of the themes which emerged, and sub sections present the categories, which include verbatim expressions upon which themes are built. Each participant’s response is coded alongside their depression assessment where “D” stands for women with depressive symptoms and “ND” for women without depressive symptoms as measured on the EPDS.

### Theme 1: purpose of life

Overall, these women were living life without specific purpose. Their goals lacked direction and they explained how they were only living their lives for their families. Many struggled to respond to the probes about their future goals - they were not certain and seemed not to have thought about this before. Their lives revolved around their husbands and children and the majority had little ambition or goals of their own. However, some women were quite clear and confident while sharing their life stories, with a spark in their eyes as they related cherished memories.

#### No personal goals

Most of the participants shared that they felt little ambition and passion towards anything, and their purpose of life was associated with their family, husband, and children. For example, three different women stated: *“Now I just think that marriage and children is the only thing left……taking care of children, and their good upbringing, this is my only purpose” (KH01- D). “To look after husband, to look after mother-in-law, to look after parents. I am doing all this and will do with Allah’s will” (GDN03- D). “The purpose of my life is that I want to see my son join the army” (KH03- ND)*.

Some participants shared clear goals for themselves, but their ultimate goal centered around others only, for example one of the participants said, *“I want to get a diploma, and this is for my children’s future.” (KA02-ND)* and another stated her life was oriented and goal directed: *“I want to spend life in a proper manner, I want life to be organized.” (KG04- ND). One participant was clear that “I want to please Allah, to offer prayers, do good deeds, so everyone can stay happy because of me” (GDN01- ND)*.

### Theme 2: dealing with emotions

Most of the participants had difficulty in expressing and managing their emotions. Their selection of words and the body language showed resistance, such that they were not able to make eye contact. A minority were able to express themselves openly and were clear and fluent in their communication. Stress was an issue for several participants who shared their anger and stress management issues and wanted to learn to manage their stress and aggression.

#### Managing stress

Few of the participants showed keen interest to learn how to manage their stress as they shared their experiences and were open to learning coping mechanisms. They were aware of their own emotions and wanted to adopt a more positive approach. Two mothers captured the essence of this theme: *“I want to deal well with stress…I want to manage the stress my parents have in their life.” (HYD02- ND)* and *“We must solve the problems that come our way. There is no point in getting tense.” (KA02- ND).*

Dealing with thoughts and emotions was an issue which emerged. The ability to comprehend feelings, to express them in words and the struggle to manage them was expressed. Anxiety was evident in their body language - one of the participants cried during the interview and many of them needed constant support from the researcher. They struggled to find words to express their worry. Two women strongly expressed the issue: *“My mind is filled with the thoughts that he will leave me some day; I am afraid of losing him” (GDN03- D). “There are some people in my in-laws who don’t behave well with me, I wondered, why did this happen to me only” (KG03- D)*.

#### Dealing with anger

Participants’ responses suggested they were struggling to manage their anger. Anger issues were evident and distressing for most of the participants and voiced clearly: *“I have a lot of bad things in me, my anger, sometimes I think due to my anger I will get into trouble too. I used to break things; I get abusive too” (GDN03- D).* Other participants stated: *“My family tells me that I am perfect in all aspects, but I get angry very easily…. I take care of everyone and that I get angry, and all my efforts get useless. My husband says, ‘that you do a lot for everyone but your anger ruins everything.” (KA02- ND).*

#### Self-harm

Some participants shared their struggle to manage their emotions, which ended in self harm. Some expressed suicidal ideas: One participant while talking about the dissatisfaction with her marital life shared that “*This shattered me so much that I wanted to die, and I even went and contemplated suicide at a bridge near Clifton.” (GDN03- D)*. The other participant also shared that, *“I harm myself and do nothing to anybody else… all anger is inside me I never take it out” (KH01- D).*

Some of the participants’ responses showed courage and readiness to address their daily life problems and tried to deal with their stressors in a positive way. The participants pointed out that *“I take everything in a positive way, I know that this thing is negative even then I try to take it as positive” (HYD02- ND).* Another demonstrated courage and stated: *“I understand that life goes on, there is no point in crying, because only I will get hurt by this. I will pressurize myself and will never come out of it. So, I must move on” (KH03- ND).*

#### Humor

Exploring ways that the participants managed with their anger and stress, revealed that some of the participants tended to use humor to deal with stressful situations. Humor helped to cope with stressful circumstances and maintain relationships with others and this was reflected in three participants’ comments: *“when I make others happy, I feel satisfied*” *(HYD 02- ND); “Making others laugh so that they feel good…. and they get peace of mind” (KH03- ND)*; *“I tell them funny things, so they laugh and in return I feel good” (KA02-ND)*.

### Theme 3: believing in yourself

When participants were asked to whom they give credit for their achievements, the majority mentioned their parents, husbands, and siblings - none of them appreciated themselves for their good deeds. It was noted that the cultural practice of not praising oneself in front of others was stopping these women from sharing their qualities and strengths. They openly talked about their flaws and drawbacks but when asked to praise themselves, were reluctant, resulting in them feeling unappreciated for their efforts.

#### Not giving credit to self

Most of the participants shared that they never give credit to themselves for their achievements as one of the participants stated that, *“I will give it to my parents” (KH01-D),* the participant also stated that she gives credit for her achievements to, *“To my husband… To my parents… They made me so capable that other people responded to me like this (positively). If they don’t make me this much capable, then others will not admire me” (KG01- D).*

On the contrary, few of the participants had confidence in themselves and their achievements and gave credit to either themselves or to God. They boldly stated that*…little to myself, some to husband and mother” (GDN-P0- ND)*. Few of them also gave credit to the divine power and did not want to praise themselves so they stated, *“I give credit to Allah. Whatever happens good or bad it’s because of him.” (KH03- ND).*

#### Self-blaming

All participants appeared to blame themselves for every mishap or failure that occurred whether this was related to her or not. As mentioned by two of the participants, *“…because of my own mistakes …. maybe something is missing in myself…. or fault in myself…. that’s why these things happen (KA01-ND)* and the other one also stated, *“…my own self, whatever happens that is wrong, it was due to me” (GDN01-ND).*

#### Self confidence

When participants were asked what is necessary in life to be strong and independent, few of them shared that confidence and valuing oneself is essential: *“Must be confident, don’t stay at the back, feel proud of yourself...don’t be bound.... Confidence is very necessary, otherwise the girl has no value” (KA03- ND)*. The other one shared that, *“I realize that a person can do everything if s/he wants.” (HYD02- ND).*

Responses from some of the participants reflected that they lacked self-confidence and courage to stand for themselves and face domestic violence further and that they seemed to struggle to voice their basic rights which has impacted on the self-image of some of the participants. As two of them quoted that, *“When it was my 17 month [of pregnancy] my husband beat me…. then after that...threw me out from home. I had my may baby at 32 weeks”. (KH01- D)* and the other participant reflected that, *“…it’s been ages since I have seen my face in mirror…. the face is not the same as it used to be…. I am not like before…. my complexion got dull” (KG03- D).*

#### Decision making power

Participants felt more empowered to make decisions before marriage. Most of the participants expressed that after marriage, they consulted their husband and in-laws in every decision. According to cultural values, when one becomes part of a family, everyone’s opinion must be considered. Upon asking this participant pointed out, *“Make decisions…but with everybody’s suggestion….by asking everyone, is this right? or that is right like this” (KA01-ND)*, *“you don’t know which decision will hurt them, so you have to share before making any decision” (KA03- ND).*

Some of the participants showed helplessness and dependency, one participant stated her desire to make decisions as, *“I want to take my own decisions myself, but I cannot” (KG03-D)*.

### Theme 4: optimistic approach

In this study participants were asked what keeps them going with life and helps them in dealing with the adversities that life brings. Most shared that hope and positivity motivates them to survive and thrive for the best. Moreover, they also expressed their optimistic approach in life due to feeling satisfied and content. When asked to list the ways they deal with life events, few participants identified positive coping strategies whereas, others lacked this ability and were not able to answer despite constant probing by the researcher. A positive attitude and evidence of resilience attributes were evident in the participants who scored low on the EPDS.

#### Being positive

Upon asking what makes them feel content and fearless in their lives, some of the participants shared that having positive mindset and ignoring the negativity helps them in dealing with stressful situations. These participants demonstrated lively personalities during the interview and stated, *“I like positivity in myself which I learnt from my father, I am broad minded. (KA03- ND)”,* and *“…don’t take things seriously. Whatever people say unnecessarily just ignore it…as I have done for some time” (KA01-ND)*.

On the contrary, most of the participants showed sadness, hopelessness, and helplessness when asked about life’s ups and downs. Two participants responded, “*While in my work life a few periods went well, few periods were full of depression…I became frustrated and obsessed” (KG-01 D)* and *“I went into depression because of my husband’s behavior” (KH-01, D).*

#### Being courageous

Most of the participants shared that being courageous and bold helped them in managing negative emotions and they said that at times one should take a stand for herself. One of the participants shared her feelings, *“when difficult times come to any person…that person showed courage…so she can easily pass that time” (KH01-D)*. The other pointed out that, *“One should have courage and strength, there is no way out if you don’t have courage, people should keep trying, there must be a way out, find a solution with courage and don’t consider yourself weak.” (GDN01-ND)*.

#### Being hopeful

Some of the participants were very hopeful regarding their future and that is what helped them in staying positive. Hope gave them light and direction to keep on moving with what life brings for them. As mentioned by one of the participants, *“one should keep trying because if you run away from the situation then that situation might convert into a big trouble which then will not be easy to deal with.” (KA03-ND)*. The other participant stated that, *“I understand that life goes on, there is no point in crying, because only I will get hurt by this. I will pressurize myself and will never come out of it…so I must move on” (KH03-ND)*.

On the other hand, few of the participants were restricted in living according to their own will, this made them feel helpless and stranded. The lack of support from family made them believe that now this is their life and they had to live this way only. As shared by few participants, *“My husband never allowed me to do any job, from the beginning I did alma [religious scholar] course, did my studies, did a beautician course, I wanted for everything, I wished that I wanted to this and that but I couldn’t” (KH01- D)* and *“A woman stays in the house, what else can she do….a woman stays at home and does home chores, it’s her duty” (KG03- D)*.

#### Happy and contented

Most of the participants shared the importance of being happy in all situations and expressed that no matter what happens one must always seek happiness in life. Participants reflected upon the importance of staying happy but few of them were not able to experience contentment or happiness. One reported, *“I try to stay happy in every situation, but situation doesn’t allow me” (KG03-D)* while another stated “*it’s a natural behavior, if you are happy today then, some other day you will feel sad too it’s a common fact” (KA03- ND).*

#### Life Goes on

Most of the participants were optimistic as they shared how ups and downs are part of life, and we must be able to deal with all the situations. They also expressed how with time everything gets better, so one must not lose hope and always try to stay positive, and that courage and being strong keeps you motivated. Two responded positively by saying, *“We must go on with life, and this is god’s will to give life and death…. Life goes on. We must move forward in life (KH03-ND),* and that *“Time heals everything” (HYD01- ND).*

### Theme 5: strengthening support and relationships

Women enrolled in this study struggled with their relationships and that created stress expressed as concerns of extra-marital affairs, abuse and lack of trust in relationships that have impacted their mental health. However, some reported sharing a good bond with their in-laws and husband.

#### Family role in maintaining relationships

Some of the participants had good relationships with their in-laws and husbands, but many others had a tumultuous and difficult relationships. Moreover, a few of the participants shared how husbands play an important role in their life and maintaining a strong relationship is important for their peace of mind. Two participants shared, *“My in-laws used to live with me, and everyone respects me. I am the youngest, but everyone respects me”* (KA02-ND) and *“this husband-and-wife relation is the strongest bond, if they are together, they can solve every problem…. If a husband falls anywhere in life, then the wife is here to hold him, same is for the husband, if the wife falls then he should hold her; they are together, a strong bond”* (KA03-ND)*.*

Most of the participants expressed a lack of support from in-laws and husband. They were struggling to have a positive relationship especially with their husbands. One of the participants stated that, *“My husband will believe others, even a child and will scold me, he never supports me” (GDN02- D)*. The other shared her sorrow as, *“My husband insulted me in front of my family members, in front of the whole society” (KH01-D).*

#### Balancing relationships

When asked to share their thoughts on how they manage their relationships effectively and the difficulties in doing so, participants shared their view on the importance of balancing relationships. Few participants stated that, *“I cannot maintain equality; my husband says this too that I cannot maintain equality among all”. (KG04-ND)*. Another participant pointed out that, *“Even my husband tells me that you are not looking after your house.” (GDN03- D)*. Considering this, researchers noticed that all participants placed an emphasis on maintaining positive relationships with all family members and taking care of domestic duties.

#### Unrealistic expectations

It became clear that unrealistic expectations are the cause of distress among relations and that not meeting the expectations creates misunderstandings. Most of the participants shared similar responses, *“My husband never allowed me to visit my parents’ house…even our homes were in front of each other….”(KH01-D), “that is the reason my husband fights, that you only get ready when you go out”.(GDN03-D)*, *“My husband once said he wants to get married again as I am not able to give him child, my heart breaks into pieces” (GDN02-D).*

### Theme 6: spirituality and humanity

Participants were able to deal with stressors more effectively due to their faith and trust in the divine power. They shared that faith in God was a major source of hope and gives them strength to deal with the stressful situations.

#### Faith & trust

When asked about spirituality, participants reflected that, *“Whenever hard times come, we go with that and Allah helps us too, it is stressful, sometimes people get hopeless, but then again, Allah, he will give you strength, he will give you a solution.” (GDN01- ND)*. Few participants shared their believes and stated that *“I believe that whatever happens, happens for good. We must stay strong, it’s upon Allah to give life and death. I trust Allah more than anyone. I always thank Allah, whenever I am in trouble, or I am in peace.” (KH03- ND).* One more participant shared that *“I take every problem as an exam as it is from Allah, you have to be faithful in this regard,” (HYD 02-ND).*

Some of the participants expressed that they trust people easily and have difficulty in analyzing the situation and staying positive. One of the participants stated her vulnerability as, *“I trusted people very easily (GDN03- D).*

#### Gratitude towards Allah

Being thankful can sometimes be a great blessing and gives internal power to women. Majority of the participants shared that showing gratitude has helped them in life. Few of the participants expressed that *“When I am alone, I just pray, Allah is merciful, he will not leave me, he will support me, Thanks to Allah many people did bad to me, but I never complained to Allah.” (GDN02-D)*, *“I always thank Allah, whenever I am in trouble, or I am in peace I thank him only”. (KH03-ND)*, *“I just thank Allah and remain patient for what I don’t have”. (HYD 02-ND).*

#### Humanity

Most of the participants expressed that by helping others, they felt more connected, contented, and competent about themselves. Further, they shared that helping others is a good deed and one must think about others as well, few participants commented that, *“According to me those people are good people who assist others, who help them in their problems (GDN01- ND), “When people get happy, they give prayers (GDN03-D)* and *“I keep others happy so that’s why it may be… No, I mean that never think bad about anyone, always think nice about everyone”. (KA02-ND)* Another participant highlighted that *“Love is the most important thing, if someone comes to you, you should give love so that the person forgets about her past while sitting with you” (GDN03-D)*.

## Discussion

Utilizing in-depth interviews this study explored the experiences and resilience attributes which enhance the prenatal mental health in a sample of 17 Pakistani women who represented women with and without depressive symptoms. The six emergent themes: purpose of life; dealing with emotions; believing in self; optimistic approach; strengthening support and relationship; and spirituality and humanity were viewed as factors enhancing resilience during stressful situations. Although, all the participants belonged to diverse background in terms of education, working status, and other factors some emerged as being less depressive compared to others. Interestingly, whether the women showed depressive symptoms or not, they voiced similar resilience attributes which they believe are important for improving their mental health.

Three recent studies conducted with pregnant women in high income countries also reported similar findings. Firstly, a study in Alberta, Canada used thematic analysis to explore the resilience practices and strategies of 54 pregnant women that supported them during traumatic experiences. These emerged as: “Relationships”; “Emotional Regulation”; and “Optimism” [60]. The results showed that remaining calm and regulating emotions helped in healing trauma, while developing a positive approach and having an optimistic outlook towards life helped participants to shift their beliefs when dealing with stressful situations [60]. Secondly, Gallagher et al. [61] in their quantitative study of 329 women in the USA found that people who are optimistic tend to adopt a positive coping strategy as compared to those who are not optimistic, and this uniquely contributes to enhancing a person’s resilience [61, 62]. Obviously, there are more components that affect the resilience of an individual but effective use of the identified attributes during pregnancy, clearly a stressful experience, is proposed to mediate an individual’s reactions and responses [63]. Hence, having a strong support system, approach and emotional regulation that deals with internal and external locus of control are all components of resilience. Thirdly, a qualitative study conducted in the USA recruited 10 mothers and pregnant women exposed to intimate partner violence and 46 service providers. Results identified that participants who had high self-esteem or positive self-perception were found to be more resilient and were able to deal with stressful situations in a more effective way. High self-esteem, the strong support of friends and family and greater empathy, forgiveness, and compassion guided them in strengthening their resilience [64]. Self-esteem as an important aspect of resilience was also articulated in a study, which was conducted among Chinese adolescents where internal factors such as self-esteem were a major source of increasing resilience in an individual [65]. I Considering previous findings from research in which more than half of pregnant women in Pakistan demonstrated low self-esteem [66], .a positive self-belief would be a key factor in building resilience.

The current study also revealed that paternal involvement and support from the paternal family kept women hopeful. Moreover, women who expressed their goals with clarity were found to be self-sufficient and self-reliant which is one of the attributes of resilience. A study conducted in southern Louisiana, USA, also shared similar findings. Despite teenage pregnancy and coming from low-income backgrounds 15 pregnant adolescents delivered healthy babies because of possessing positive factors such as self-efficacy, motivation to achieve career goals, and having a strong support system, which the authors referred to as a resiliency framework [67]. In Pakistan, however, the majority of women belong to patriarchal family systems, lack participation in domestic decision making, lack autonomy, and with illiteracy this leads to indecisiveness, poor self-confidence, and prenatal depression, culminating in low resilience [68].

Moreover, Payne [69] in a study conducted on teenage pregnant women outlined similarly consistent findings, indicating that individual characteristics of resilience compatible with different theoretical perspectives included, sense of purpose/meaning of life, optimism, representation of relationships, personal efficacy, self-regulation, sense of humor, self-perception, and hopefulness. To be resilient in stressful situations one must have a goal and direction that helps in restoring faith and provides a sense of meaning in life [70].

Support systems and relationships were found to be associated with resilience. Participants who had less depressive symptoms had the support of family or friends and they referred to this support as their biggest strength. A meta-analysis of 120 studies, conducted by Pilkington et al. [7] suggested that relationship satisfaction is one of the strongest protective factors against perinatal depression. On the other hand, poor communication, conflict, and dissatisfaction with the partner relationship can increase the risk of depression during the transition to parenthood [7]. The bond that pregnant women share with their spouse during pregnancy contributes to prenatal mental health and stress [71] and ensuring healthy marital relationship which is vital factor in enhancing resilience.

Another cross-sectional study conducted on 122 primiparous women also supported the notion that the main elements of resilience for reducing stress is self-confidence, optimism, positive acceptance of change, and spiritual influences [37]. Consistent results were also identified in another study where 10 pregnant women and 46 service providers were interviewed via focus groups about the personal strengths of women exposed to intimate partner violence (IPV). The results showed that increased resilience was associated with spirituality, sense of humor, and hope [64]. Faith and trust in the divine power can be a great source of motivation for those who lose hope and spirituality and resilience has been proven to be associated with each other [72]. Additionally, a prominent resilience attribute in this study was ‘Spirituality and Humanity,’ - individuals who had a strong faith in God, practiced gratitude, and were optimistic were able to cope better with stressful conditions. It was clear that humanity and spirituality were important factors in fostering and strengthening resilience among resilient participants [73].

On the other hand, Kishore [74] reported that resilience alone is not sufficient to protect women from depression during pregnancy. It should be moderated by social support particularly from friends, families, and relatives [74]. This was also identified in the current study where strengthening support systems was also found to be an attribute for enhancing resilience.

In view of the adverse impact of depression during pregnancy on the lives of women and their offspring it behooves the health care professions to develop resilience building intervention [75] which may prevent adverse mental and physical outcomes. This concept was promulgated in a study in China with 605 pregnant women which identified prenatal depression as negatively associated with resilience [76]. Hence improving women’s resilience through resilience-enhancement training can reduce the detrimental effects of pregnancy and improve women’s mental wellbeing [76]. This was also reported in a study on pregnant women in Spain, which proposed that resilience training should be introduced to improve psychological health of pregnant women and their infants [36].

The six emergent themes are well supported by closely related themes in the literature, including leading a purposeful life [67, 77], emotional management [78], optimism [78, 79], ,self-belief [77], positive relationships [64, 79] and spiritual support [79, 80].

An exploration of resilience attributes through the ecological system framework utilized in this study help researchers to conceptualize the proximal and distal factors that can predict successful development of an intervention. The literature also support the adaptation of a a multisystemic social-ecological theory to offers a perspective on intervention that focuses on variables that can be changed [81]. Hence, these six identified themes will surely aid in developing and evaluating resilience-building interventions that are culturally and contextually appropriate and can be scaled up across the country to demonstrate its ability to reduce prenatal depression. The researchers believe that it is crucial to provide perinatal women the skills which is person-centered, tailored to individual needs, and efficient in terms of both time and money. Future intervention will be designed with a focus on a highly individualized, accessible, and culturally acceptable strategies, whose advantages can be felt throughout pregnancy, parenthood, and beyond.

### Strengths and limitations

Current study is the first to explore the experiences and resilience attributes of pregnant women in Pakistan. Although coming from different language and subcultural backgrounds, the sample were heterogeneous in terms of age, gestational age, education, and working status. To ensure rigor, the study used a variety of techniques. The data collection was well-organized, and interviews were done by a researcher with extensive experience in the relevant field, maintaining credibility. Additionally, the data organization and condensation processes of the data reduction and analysis steps ensured quality data where emergent themes covered the core data. To keep the confirmability, a detailed data analysis method was used, including individual researcher coding and recoding of whole datasets, followed by a group discussion of similarities and differences. Transferability was ensured by choosing participants from a variety of backgrounds to reflect the diversity of the Pakistani setting. Using purposive sampling to form a preferred sample can also assure transferability [82]. Moreover, to make the methodology reproducible by other researchers, a detailed explanation of the setting, phases of data analysis, and data gathering procedures were also provided. Trained research assistant recorded field notes and saved all raw data, observations, and reflections to maintain reliability. Furthermore, ensuring dependability, the procedure of data collecting, transcription, and analysis was finished within a predetermined time frame [83, 84]. Moreover, we continued recruiting participants until saturation achieved in findings. This further improves the validity of the finding [46, 85].

The limitations include firstly that the EPDS depression scores that reflect the last 15 days, might not reflect participants’ overall experiences of depression, compared to the semi-structured interview which provided a broader view of their life experiences. Secondly the study cannot be generalized to the population of Pakistan as the study only recruited a limited number of women coming from lower-socio economic backgrounds, and in keeping with research of this nature, the sampling was neither random nor representative, yet enabled rich data gathered from the small sample with the lived experience.

## Conclusion

The six themes and attributes which emerged from the experiences of these participants provide a pathway for understanding and helping develop resilience and provide a foundation for future intervention studies. To promote mental well-being during pregnancy, in the context of a women experiencing pregnancy in a lower socioeconomic stratum in Pakistan, valid resilience building interventions that are culturally and contextually appropriate will be designed based on these findings and the language of the participants. This will not only improve the mental status of pregnant women but also their children and families.

## Data Availability

The datasets generated and/or analyzed during the current study are not publicly available because the dataset contains interview transcripts, which may compromise participant privacy and confidentiality due to the sensitive nature of the topics, but are available from the corresponding author on reasonable request.

## Abbreviations

LMICs: Low- and middle-income countries
UK: United Kingdom
USA: United States of America
AKUH: Aga Khan University Hospital
KG: Koohi Goth
EPDS: Edinburgh Postnatal Depression Scale
PI: Principal Investigator
KH: Kharadar
GDN: Garden
KA: Karimabad
HYD: Hyderabad
